# Valorisation of Sea Buckthorn Pomace by Optimization of Ultrasonic-Assisted Extraction of Soluble Dietary Fibre Using Response Surface Methodology

**DOI:** 10.3390/foods10061330

**Published:** 2021-06-09

**Authors:** Shehzad Hussain, Minaxi Sharma, Rajeev Bhat

**Affiliations:** ERA-Chair for Food (By-) Product Valorisation Technologies (VALORTECH), Estonian University of Life Sciences, Fr.R. Kreutzwaldi 56/5, 51006 Tartu, Estonia; minaxi.sharma@emu.ee (M.S.); rajeev.bhat@emu.ee (R.B.)

**Keywords:** waste valorisation, sea buckthorn, soluble dietary fibre, response surface methodology, hydration properties

## Abstract

Sea buckthorn pomace is a valuable industrial waste/by-product obtained after juice production that contains bioactive, health-promoting dietary fibres. This pomace finds usage as animal feed or simply discarded, owed to the lack of appropriate handling or processing facilities. The present study was aimed to evaluate the effects of green extraction technologies such as ultrasonic-assisted extraction on the yield of soluble dietary fibre (SDF) from sea buckthorn pomace. Response surface methodology (RSM) coupled with Box–Behnken design (BBD) was applied for optimization of SDF yield. The effects of sonication temperature (60–80 °C), sonication power (100–130 W) and extraction time (30–60 min) on the yield of SDF were also investigated. Furthermore, colour measurement and hydration properties of sea buckthorn pomace powder (STP) and dietary fibre fractions (SDF and insoluble dietary fibre, IDF) were also investigated. From the RSM results, the optimal sonication temperature (67.83 °C), sonication power (105.52 W) and extraction time (51.18 min) were identified. Based on this, the modified optimum conditions were standardised (sonication temperature of 70 °C, sonication power of 105 W and extraction time of 50 min). Accordingly, the yield of SDF obtained was 16.08 ± 0.18%, which was close to the predicted value (15.66%). Sonication temperature showed significant effects at *p* ≤ 0.01, while sonication power and extraction time showed significant effects at *p* ≤ 0.05 on the yield of SDF. The result on colour attributes of STP, SDF and IDF differed (L* (STP: 54.71 ± 0.72, IDF: 72.64 ± 0.21 and SDF: 54.53 ± 0.31), a* (STP: 52.35 ± 1.04, IDF: 32.85 ± 0.79 and SDF: 43.54 ± 0.03), b* (STP: 79.28 ± 0.62, IDF: 82.47 ± 0.19 and SDF: 71.33 ± 0.50), and ∆E* (STP: 79.93 ± 0.50, IDF: 74.18 ± 0.30 and SDF: 68.40 ± 0.39)). Higher values of hydration properties such as the water holding, swelling and oil holding capacities were found in SDF (7.25 ± 0.10 g g^−1^, 7.24 ± 0.05 mL g^−1^ and 1.49 ± 0.02 g g^−1^), followed by IDF (6.30 ± 0.02, 5.75 ± 0.07 and 1.25 ± 0.03) and STP (4.17 ± 0.04, 3.48 ± 0.06 and 0.89 ± 0.03), respectively. Based on our results, response surface methodology is recommended to be adopted to optimize the ultrasonic-assisted extraction to obtain maximum yield of SDF from sea buckthorn pomace. These results can be of practical usage while designing future functional food formulations using sea buckthorn pomace.

## 1. Introduction

Sea buckthorn (*Hippophae rhamnoides L*.) is abundantly cultivated in various parts of the world including China, India, Russia and many parts of Northern Europe. This crop sustains hard growing conditions such as salty, sandy and dry conditions [1]. It is generally cultivated to tap the proved potentiality/usage in food and health sectors. In the growing regions, traditionally sea buckthorn berries are used to treat the common cold, cough, intestinal disorders, etc. Some of the food preparations include production of juice, jellies, jams, oil and dietary supplements, as well as use in cosmetics [2]. Sea buckthorn’s fruit, leaves and seeds are an abundant source of numerous bioactive compounds [3,4,5]. These are rich in essential nutrients and bioactive compounds. Sea buckthorn grown in India and Sweden have been reported to show the presence of sterols, polyphenolic compounds, tocopherols and carotenoids [6]. Besides, sea buckthorn fractions with low polarity have the ability to eliminate cancer cells [7]. Some of the potential therapeutic activities exerted by sea buckthorn include anti-diabetic, anti-cancer, anti-inflammatory, antimicrobial and antioxidant effects. Besides, they are effective in reducing the risk of cardiovascular diseases, stomach ulcers and skin disease [8]. Bioactive compounds extracted from sea buckthorn have been fortified in some of the bakery-based products. These products were reported to hinder the starch digestion, which is beneficial for the population with diabetes [9].

Fruits and vegetable wastes/by-products of the dependent processing industries are considered to be an underutilised resource and can be efficiently valorised to obtain value-added products [10]. With regard to sea buckthorn, after processing of the berries (to obtain juice, jam and jellies), huge amounts of processing wastes (pomace) are produced. This pomace finds usage as animal feed or is simply discarded, owed to the lack of appropriate handling or processing facilities. As a consequence, annually significant amounts of nutrients are untapped and lost [11]. The development of extraction techniques have validated the valorisation of pomace from fruits like sea buckthorn [12,13], raspberry [14,15], black chokeberry [16] and black currant [17].

Fruit processing wastes/by-products are considered to be a hidden source of bioactive compounds, including phenolics, carotenoids and dietary fibres, etc. [18]. Dietary fibres obtained from these wastes/by-products can be a potential source of functional dietary constituent, which can find potential applications in food and pharmaceutical industries as a reliable inexpensive raw material to produce value-added food products [10]. Many researchers have discovered that dietary fibre intake is linked to decreasing the risk of cardiovascular diseases [19], cancer [20], obesity [21] and diabetes [22].

On the other hand, effective application of optimization techniques like that of Response surface methodology (RSM) in the course of extraction might be alluring due to a lesser experimental trials requirement than that of a single-factor method. Moreover, RSM is capable of identifying interactions among various variables by establishing an applicable mathematical model/equation [23]. As per our knowledge, there are no studies conducted on sea buckthorn pomace to optimize the extraction yield of dietary fibres by an ultrasound-assisted extraction technique using RSM. Therefore, an attempt was made to identify/enhance the yield of dietary fibre by ultrasound-assisted extraction from sea buckthorn pomace by adopting three independent variables: sonication temperature (60–80 °C), sonication power (100–160 W) and extraction time (30–60 min). Moreover, in this study, techno-functional or hydration characteristics such as water holding capacity (WHC), swelling capacity (SWC) and oil holding capacity (OHC) were also determined. The outcome of the present study is expected to provide evidence for the valorisation of sea buckthorn pomace to obtain functional ingredients such as dietary fibre that can find potential applications while producing value-added products. 

## 2. Materials and Methods

### 2.1. Material and Reagents

Sea buckthorn pomace was obtained from the Polli Horticulture Research Centre of Estonian University of Life Sciences, Viljandi County, Estonia. After removal of seeds, the sample were freeze-dried, ground and packaged in zipped airtight polythene bags. The freeze-dried powder of sea buckthorn pomace was stored at refrigeration temperature (4–7 °C) until further analysis. All reagents used in this study were of analytical grade.

### 2.2. Extraction of Dietary Fibre from Sea Buckthorn Pomace

The ultrasound-assisted extraction of soluble dietary fibre (SDF) from freeze-dried sea buckthorn pomace was carried out using Digital Sonifier^®^ S450 CE attached with a 13 mm diameter disruptor horn (400 W Power, 20 kHz Frequency; Branson Ultrasonics Co., Danburry, CT, USA).

Previously reported methods of Liew et al. [24] and Wang et al. [25] were followed, with slight modification for the extraction process. Briefly, freeze-dried sea buckthorn powder was dispersed in 80 °C of 0.1 N citric acid solution, at a liquid:solid ratio of 25:1 (mL g^−1^), and mixed with the help of a magnetic stirrer. This mixture of extraction sample was then incubated at 80 °C for 1 h in a water bath with continuous magnetic stirring.

The glass beaker containing the extraction sample was then placed in an ultrasonic extractor chamber. Extractions were done at different sonication temperatures (60, 70 and 80 °C) for different time durations (30, 45 and 60 min) at a sonication power of 100, 130 and 160 W, respectively.

The samples were then centrifuged at 6000× *g* for 10 min and the supernatant was separated and precipitated with double the volume of ethanol (95%, 1:2 *v*/*v*). The sample mixture was then filtered under vacuum filtration with Whatman No. 1 filter paper. Filtrate was then separated, washed twice with 75% ethanol, freeze-dried and kept in desiccator at room temperature (25 ± 2 °C) prior to analysis.

### 2.3. Yield Determination

After extraction and drying process, the yield (%) of SDF was calculated as follows using Equation (1):

(1) $$\mathrm{Yield}\;\mathrm{of}\;\mathrm{soluble}\;\mathrm{dietary}\;\mathrm{fibre}\;\left(\%\right)=\frac{\mathrm{Weight}\;\mathrm{of}\;\mathrm{extracted}\;\mathrm{fibre}\;}{\mathrm{Sample}\;\mathrm{weight}}\times 100$$

### 2.4. RSM Design for Optimization of Extraction Yield

A three-level, three-factor RSM combined with Box–Behnken design (BBD) was used to examine the best order of variables to get the maximum yield of SDF from sea buckthorn pomace. Table 1 shows the coded levels and range of independent variables. The parameters including sonication temperature (°C), sonication power (W) and extraction time (min) were selected according to the results of single-factor experiment and were labelled as *X*1, *X*2 and *X*3. The yield (%) was taken as the response of the designed experiment. Table 2 lists the complete design of experiments, including 17 runs with independent variables at three variant levels.

### 2.5. Water Holding Capacity (WHC)

WHC of sea buckthorn pomace powder (STP) and dietary fibre samples was analysed in triplicate by adopting a previously used method by Raghavendra et al. [26]. Briefly, a known amount of the sample (1 g) was weighed into a graduated test tube. Distilled water (30 mL) was added and samples were then dehydrated at room temperature (25 ± 2 °C) for 18 h. The supernatant was then discarded by passing it through a sintered glass crucible under vacuum. The hydrated weight was noted and samples were then dried at 105 °C to obtain the residual dry weight using the following Equation (2):

(2) $$\mathrm{WHC}\;({g\;g}^{-1})=\frac{\mathrm{Wet}/\mathrm{hydrated}\;\mathrm{weight}-\mathrm{Dry}\;\mathrm{weight}\;\mathrm{of}\;\mathrm{sample}}{\mathrm{Dry}\;\mathrm{weight}\;\mathrm{of}\;\mathrm{sample}}$$

### 2.6. Swelling Capacity (SWC)

SWC of STP and dietary fibre fractions (Soluble dietary fibre, SDF and Insoluble dietary fibre, IDF) was measured by using the method of Raghavendra et al. [26]. A precisely weighed sample (2 g) taken in a test tube was mixed with distilled water (10 mL) and left overnight without disturbance. The final volume reached by samples was detected using Equation (3) given below:

(3) $$\mathrm{SWC}\;\left({\mathrm{mL}\;g}^{-1}\right)=\frac{\mathrm{Volume}\;\mathrm{occupied}\;\mathrm{by}\;\mathrm{the}\;\mathrm{hydrated}\;\mathrm{samples}}{\mathrm{Initial}\;\mathrm{sample}\;\mathrm{weight}}$$

### 2.7. Oil Holding Capacity (OHC)

OHC of STP, IDF and SDF was quantified by using the previously described methods of Sangnark and Noomhorm [27] with slight modifications. In a centrifuge tube, an accurately weighed sample (5 g) was mixed with rapeseed oil (Oilio, EMB Grupp AS, Tallinn, Estonia) and left for 1 h at room temperature (25 ± 2 °C). This was followed by centrifugation at 1500× *g* for 10 min. The supernatant obtained after centrifugation was separated from the pellet by filtration. OHC was calculated using the following Equation (4):

(4) $$\mathrm{OHC}\;({g\;g}^{-1})=\frac{\mathrm{Pallet}\;\mathrm{weight}-\mathrm{Dry}\;\mathrm{weight}\;\mathrm{of}\;\mathrm{sample}}{\mathrm{Dry}\;\mathrm{weight}\;\mathrm{of}\;\mathrm{sample}}$$

### 2.8. Colour Analysis

The surface colour values of STP, IDF and SDF were measured using the X-Rite 964 spectrophotometer (RPimaging INC, Grand Rapids, MI, USA), and expressed as L* (L* = 0 (black) and L* = 100 (white)), a* (−a* = greenness and +a* = redness) and b* (−b* = blueness and +b* = yellowness) (X-Rite Model: 964). The X-Rite 964 spectrophotometer (RPimaging INC, Grand Rapids, MI, USA) was used as a standard reference in numerous studies [28,29,30] in order to evaluate the colour of various food samples as L*, a*, b* and ∆E* values. Triplicate values were taken for each set of samples and the colour difference among dietary fibre samples was calculated using the following Equation (5) [31]:

(5) $$\Delta E^{*}=\sqrt{\Delta L^{*2}+\Delta a^{*2}+\Delta b^{*2}}$$

### 2.9. Statistical Analysis

Design Expert 12 software (Stat-Ease Inc., Minneapolis, MN, USA) was used for statistical analysis to determine the obtained yield (%), while the results of hydration properties and colour analysis were statistically analysed with origin Pro 8.0 (Origin Lab, Northampton, MA, USA). Obtained results were represented as means ± SD, and the data were analysed by employing one-way analysis of variance (ANOVA), followed by LSD test. 

## 3. Results and Discussion 

### 3.1. Optimization of Ultrasound-Assisted Extraction of SDF

An ultrasonic-assisted extraction technique was selected to optimize the yield of SDF from sea buckthorn pomace. The number of independent variables that can generally affect the process were identified to be the extraction temperature, sonication time and amplitude, sample to solvent ratio, solvent composition, particle size of sample, vessel shape and horn size [32]. In the present study, the independent variables selected for the optimization of the extraction process included ultra-sonication temperature, amplitude/power and extraction time. RSM was used to generate a prediction model to optimize the conditions of extraction of SDF from sea buckthorn pomace. The coded values and corresponding results with predicted values and the design matrix of RSM experiments were used to evaluate the effects of the three independent variables on the yield. This is explicated in Table 2.

Further, the model summary for yield is provided in Table 3. Data obtained showed that the quadratic model was the best fit for yield of SDF based on *p* value of the model, lack of fit and R^2^ value. Previous studies described that a significant (*p* ≤ 0.05) *p* value and an insignificant (*p* ≥ 0.05) lack of fit with R^2^ value (at least 0.80) is suitable to fit into the model [33,34]. 

In total, 17 runs were performed to optimize each parameter in the BBD design. Besides, in order to acquire a regression equation that might predict the response (*y*), the dependent and independent variables were analysed. The mathematical equation describing the extraction yield (%) of SDF as a function of the test independent variables was given by following quadratic Equation (6) in terms of coded values:

(6) $$y=16.94-1.89X1+0.86X2-0.46X3+0.49X1X2+0.12X1X3-0.61X2X3-0.61X1^{2}-1.03X2^{2}-2.13X3^{2}$$

**Figure 1:**
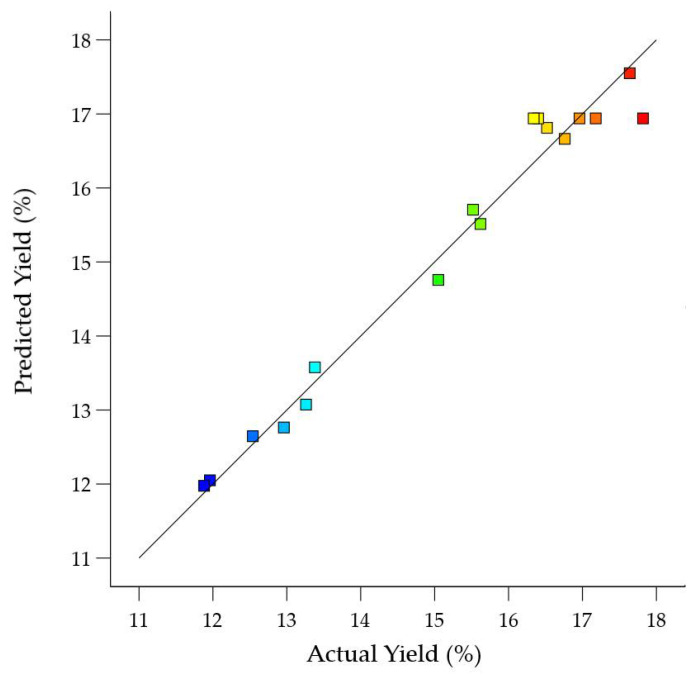
Correlation of actual and predicted yield of soluble dietary fibre (%). Blue colour indicates the lowest, while red colour shows the highest actual and predicted yields (%) of soluble dietary fibre.

In the above equation, ‘*y*’ represents the yield of SDF, while *X*1, *X*2 and *X*3 are sonication temperature (°C), sonication power (W) and extraction time (min), respectively. A satisfactory fit of plot for experimental and actual value of SDF yield was shown in Figure 1. It was observed that the regression model was a significant model with an R^2^ value of 0.9373 and a non-significant lack of fit value of 0.801. The quadratic regression model indicated that R^2^ and adjusted R^2^ were 0.9726 and 0.9373, which indicated that 97.26% of the variation could be represented by the fitted model. The R^2^ value (0.9373) suggested that the fitted model could explain 93.73% of the variations (Table 4).

As shown in Table 4, the model F-test implied that it had a higher F-value of 27.56 with a very small *p*-value of *p* ≤ 0.0001. This indicates that the model generated was significant and could characterise the relationship between parameters well. The significance of each individual coefficient was assessed using F and *p*-values. In this model, the three linear terms (*X*1, *X*2 and *X*3) and one interaction term (*X*2*X*3) showed a significant effect on the yield of SDF. The quadratic terms *X*1^2^, *X*2^2^ and *X*3^2^ were also important factors in the yield of SDF from sea buckthorn pomace.

The results elucidated in Table 4 clearly indicate that extraction conditions (temperature, power and time) significantly (*p* < 0.05) affect the yield of SDF. Previously, Chen et al. [35] reported similar results for ultrasonic-assisted extraction of dietary fibre from peels of citrus fruit. Another study conducted by Wang et al. [25] also reported the significant effects of sonication power and time on the yield of dietary fibre from corn pericarp. 

### 3.2. Response Surface Analysis

**Figure 2:**
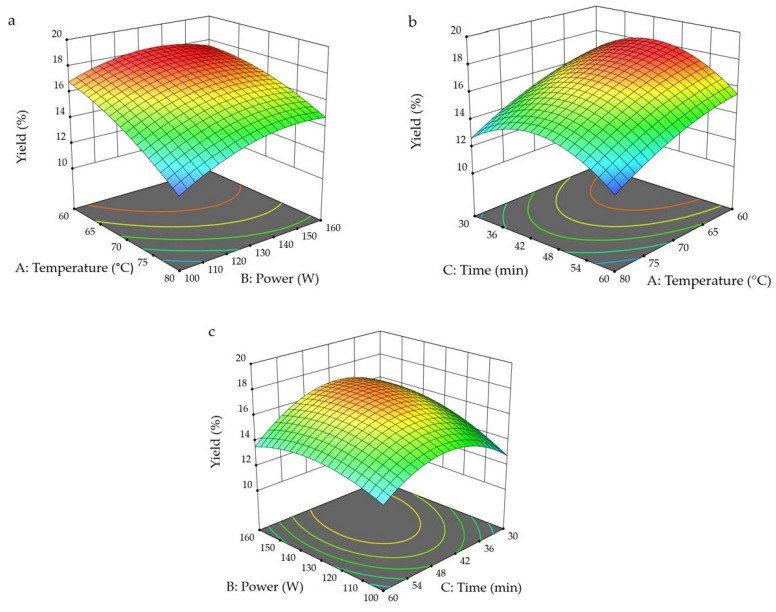
Three-dimensional response surface plots (**a**) showing the effects of ultrasonic temperature and power, (**b**) showing the effects of ultrasonic temperature and time and (**c**) showing the effect of ultrasonic power and time on soluble dietary fibre yield. Blue colour indicates the lowest, while red colour shows the highest yields (%) of soluble dietary fibre.

The three-dimensional (3D) response surface plots were produced by the model (see Figure 2a–c) to show the visual effects of experimental variable levels on the response, relationship or interaction between two independent variables as well as to evaluate the optimal level of variables for optimized yield of SDF from sea buckthorn pomace. In each 3D plot surface, two variables were presented and other variables were fixed at 0 level. It is obvious from the 3D plot (Figure 2a) that sonication temperature showed a higher significant influence compared to sonication power and extraction time. The interactions between sonication temperature and extraction time, and sonication power and extraction time are elucidated in Figure 2a–c, respectively.

### 3.3. Optimization of Parameters and Verification of the Model

The results obtained from numerical optimization revealed that the optimized conditions were sonication temperature of 67.83 °C, sonication power of 105.52 W and extraction time of 51.18 min. Under the optimal conditions, the maximum yield of SDF predicted by the model was 15.66%. Nevertheless, considering the operability for actual extraction, the optimum conditions could be altered to a sonication temperature of 70 °C, sonication power of 105 W and extraction time of 50 min. In order to link the results of predicted yield with the results of experimental yield, triplicate extraction of SDF was carried out at modified optimum conditions. Under the modified extraction conditions, the yield (experimental) of SDF was recorded as 16.08% ± 0.18, which was close to the predicted yield of SDF. As a result, BBD design was found to be precise and a significant tool for predicting the yield of dietary fibres from sea buckthorn pomace by means of a green extraction technique such as ultrasonic-assisted extraction.

### 3.4. Hydration Properties and Colour Evaluation of Sea Buckthorn Pomace Dietary Fibres

Hydration characteristics including WHC, SWC and OHC of STP and dietary fibre fractions (IDF and SDF) extracted from freeze-dried sea buckthorn pomace under modified optimized conditions (sonication temperature of 70 °C, sonication power of 105 W and extraction time of 50 min) were also evaluated and the results are presented in Table 5. Hydration properties such as SWC of various dietary fibre fractions exposed its levels of ideal application for desired texture in numerous food products and use for beneficial physiological and functional characteristics [36]. 

All of the three samples indicated a significant (*p* ≤ 0.05) difference in the values of hydration characteristics. Highest values for WHC, SWC and OHC were recorded in the SDF sample [(WHC 7.25 ± 0.10 (g g^−1^), SWC 7.24 ± 0.05 (mL g^−1^) and OHC 1.49 ± 0.02 (g g^−1^)), followed by IDF (WHC 6.30 ± 0.02 (g g^−1^), SWC 5.75 ± 0.07 (mL g^−1^) and OHC 1.25 ± 0.03 (g g^−1^)), while the lowest values for hydration characteristics were found in the STP sample (WHC 4.17 ± 0.04 (g g^−1^), SWC 3.48 ± 0.06 (mL g^−1^) and OHC 0.89 ± 0.03 (g g^−1^)]. Earlier, Kurek et al. [37] studied the hydration properties of dietary fibres extracted from cereals and reported similar results for WHC and OHC. The results of the present research work are also in agreement with the findings of Zhu et al. [38] who studied the hydration attributes of dietary fibres extracted from buckwheat hulls. The results are also in agreement with the reports of Huang and Ma [39] who investigated the physicochemical properties water-soluble dietary fibre from sweet orange pomace and Tejada-Ortigoza [40] who studied the dietary fibre from orange peel.

While determining the acceptability of food products, colour plays a vital role. Any variations in the colour may also affect the sensory properties of the samples [36]. Light scattering and absorbance are different phenomena that can contribute to the colour of a material [41]. In sea buckthorn, carotenoids can absorb light, whereas any variation in the particle size can cause light scattering [42]. The results of colour attributes such as L*, a*, b* and ∆E* of STP and dietary fibre fractions (SDF and IDF) obtained under modified optimized conditions (sonication temperature of 70 °C, sonication power of 105 W and extraction time of 50 min) in this study are elucidated in Table 5. All of the three samples showed variations in the colouring values for L* (STP: 54.71 ± 0.72, IDF: 72.64 ± 0.21 and SDF: 54.53 ± 0.31), a* (STP: 52.35 ± 1.04, IDF: 32.85 ± 0.79 and SDF: 43.54 ± 0.03), b* (STP: 79.28 ± 0.62, IDF: 82.47 ± 0.19 and SDF: 71.33 ± 0.50) and ∆E* (STP: 79.93 ± 0.50, IDF: 74.18 ± 0.30 and SDF: 68.40 ± 0.39). ∆E* value shows the change of colour among samples, and this was a light orange/yellow colour, which might be attributed to a higher content of carotenoids in the sea buckthorn pomace. A significant (*p* ≤ 0.05) difference was also noticed in the colour among all three samples studied. Previously, Yan and Kerr [43] investigated the colour of vacuum-belt and freeze-dried samples of apple pomace powders, while Bendar et al. [44] assessed the colour of grape pomace skin flour. However, there are no specific studies available on the colour assessment of dietary fibres extracted from pomace of fruits.

## 4. Conclusions

In the present study, yield of SDF was optimized by an ultrasound-assisted extraction method by applying a three-level, three-factor RSM methodology engaged with BBD. Optimum conditions for extraction were determined based on response surface methodology. At the modified optimized variables (sonication temperature of 70 °C, sonication power of 105 W and extraction time of 50 min), by using RSM the optimal yield of SDF was 16.08%, which was much close to the predicted yield. Moreover, improved hydration properties were also recorded in the SDF sample with significant colour changes in STP and dietary fibre fractions (IDF and SDF). It is expected that the results generated from this study will be of practical help to provide evidence for effective valorisation of sea buckthorn pomace to obtain dietary fibre, which can find potential applications in producing novel, value-added products.

## Figures and Tables

**Table 1:** Independent variable values with their corresponding levels used in the process.

Independent Variables	Symbol		Levels		
	Un-Coded	Coded	−1	0	1
**Ultrasonic temperature (°C)**	*X*1	*X*1	60	70	80
**Ultrasonic power (W)**	*X*2	*X*2	100	130	160
**Ultrasonic time (min)**	*X*3	*X*3	30	45	60

**Table 2:** Box–BehnkeBox–Behnken Design (BBD) design with observed responses and predicted values for the yield of soluble dietary fibre from sea buckthorn pomace.

	Coded Variable Levels			Yield of Soluble Dietary Fibre (%)	
Run	*X*1	*X*2	*X*3	Actual Yield	Predicted Yield
**1**	−1 (60)	0 (130)	1 (60)	15.62	15.51
**2**	0 (70)	−1 (100)	−1 (30)	12.96	12.76
**3**	0 (70)	1 (160)	−1 (30)	15.52	15.71
**4**	−1 (60)	0 (130)	−1 (30)	16.76	16.66
**5**	0 (70)	0 (130)	0 (45)	16.40	16.94
**6**	−1 (60)	−1 (100)	0 (45)	16.52	16.81
**7**	0 (70)	0 (130)	0 (45)	16.96	16.94
**8**	1 (80)	0 (130)	−1 (30)	12.54	12.65
**9**	0 (70)	0 (130)	0 (45)	17.82	16.94
**10**	0 (70)	0 (130)	0 (45)	16.34	16.94
**11**	0 (70)	1 (160)	1 (60)	13.38	13.58
**12**	0 (70)	−1 (100)	1 (60)	13.26	13.07
**13**	1 (80)	−1 (100)	0 (45)	11.96	12.05
**14**	0 (70)	0 (130)	0 (45)	17.18	16.94
**15**	−1 (60)	1 (160)	0 (45)	17.64	17.55
**16**	1 (80)	0 (130)	1 (60)	11.88	11.98
**17**	1 (80)	1 (160)	0 (45)	15.05	14.76

**Table 3:** Model summary of yield.

Response	Source	Sum of Squares	df	Mean Square	F-Value	Model *p*-Value	Lack of Fit	R^2^
	**Mean**	3909.16	1	3909.16				
**Yield (%)**	**Linear**	36.13	3	12.04	4.96	0.0165 *	0.0244 *	0.4259
	**2FI**	2.52	3	0.8387	0.2885	0.8328	0.0146 *	0.3131
	**Quadratic**	27.22	3	9.07	34.16	0.0001 **	0.80	0.9373

Significant at * p < 0.05. Significant at ** *p* < 0.01.

**Table 4:** One-way Analysis of variance (ANOVA) of model for the yield of soluble dietary fibre from sea buckthorn pomace.

Source	Sum of Squares	df	Mean Square	F-Value	*p*-Value
Model	65.86	9	7.32	27.56	0.0001 **
A	28.54	1	28.54	107.47	<0.0001 **
B	5.93	1	5.93	22.35	0.0021 **
C	1.66	1	1.66	6.24	0.0411 *
AB	0.9702	1	0.9702	3.65	0.0975
AC	0.0576	1	0.0576	0.2169	0.6555
BC	1.49	1	1.49	5.61	0.0498 *
A^2^	1.59	1	1.59	5.97	0.0445 *
B^2^	4.5	1	4.5	16.94	0.0045 **
C^2^	19.04	1	19.04	71.68	<0.0001 **
Residual	1.86	7	0.2655		
Lack of Fit	0.3748	3	0.1249	0.3368	0.801
Pure Error	1.48	4	0.371		
Cor Total	67.72	16			
C.V%	3.4				
R^2^	0.9726				
Adjusted R^2^	0.9373				

Significant at * p < 0.05. Significant at ** *p* < 0.01.

**Table 5:** Hydration properties and colour evaluation of dietary fractions of sea buckthorn pomace.

Sample	Hydration Properties			Colour Measurement			
	WHC (g g^−1^)	SWC (mL g^−1^)	OHC (g g^−1^)	L*	a*	b*	∆E*
STP	4.17 ± 0.04 ^c^	3.48 ± 0.06 ^c^	0.89 ± 0.03 ^c^	54.71 ± 0.72 ^b^	52.35 ± 1.04 ^a^	79.28 ± 0.62 ^b^	79.93 ± 0.50 ^a^
IDF	6.30 ± 0.02 ^b^	5.75 ± 0.07 ^b^	1.25 ± 0.03 ^b^	72.64 ± 0.21 ^a^	32.85 ± 0.79 ^c^	82.47 ± 0.19 ^a^	74.18 ± 0.30 ^b^
SDF	7.25 ± 0.10 ^a^	7.24 ± 0.05 ^a^	1.49 ± 0.02 ^a^	54.53 ± 0.31 ^b^	43.54 ± 0.03 ^b^	71.33 ± 0.50 ^c^	68.40 ± 0.39 ^c^

WHC—water-holding capacity; SWC—swelling capacity; OHC—oil-holding capacity; STP—sea buckthorn pomace powder; IDF—insoluble dietary fibre; SDF—soluble dietary fibre; L*—colour brightness; a*—colour in the range from green to red; b*—colour from blue to yellow; ∆E* —total colour difference between samples. The values are presented as mean ± SD (n = 3). Mean values were compared by employing LSD test. The values followed by different superscript letters (a–c) within the same column are significantly different *(p* ≤ 0.05) from each other.

## Data Availability

Not applicable.
